# Efficacy of afatinib for pulmonary adenocarcinoma with leptomeningeal metastases harboring an epidermal growth factor receptor complex mutation (exon 19del+K754E): A case report

**DOI:** 10.1097/MD.0000000000022851

**Published:** 2020-10-23

**Authors:** Chunhua Ma, Mei Liu, Ning Mu, Jinduo Li, Lin Li, Rong Jiang

**Affiliations:** Department of Intervention, Tianjin HuanHu Hospital, Tianjin Key Laboratory of Cerebral Vascular and Neurodegenerative Diseases, Tianjin, China.

**Keywords:** afatinib, cerebrospinal fluid, EGFR exon19del+K754E complex mutation, leptomeningeal metastasis, NSCLC

## Abstract

**Rationale:**

Liquid biopsy of cerebrospinal fluid (CSF) and sequencing of cell-free DNA has rarely been used to identify epidermal growth factor receptor (EGFR) mutations, which can guide the design of precise, personalized treatment for patients with leptomeningeal metastasis from lung adenocarcinoma.

**Patient concerns:**

A 42-year-old woman with lung adenocarcinoma and leptomeningeal metastasis was admitted to our hospital on March 31, 2019. She exhibited no response to treatment with gefitinib, osimertinib, or chemoradiotherapy and was in critical condition, with an expected survival of <4 weeks.

**Diagnosis:**

Next-generation sequencing of CSF and peripheral blood samples identified an EGFR complex mutation (exon19del+K754E).

**Interventions:**

On April 10, 2019, the patient started oral afatinib (40 mg po qd), but she developed a grade III oral mucosal reaction 1 week later. The afatinib dose was reduced to 30 mg po qd.

**Outcomes:**

At the follow-up examination on May 15, 2019, the patient reported relief from headaches. Enhanced magnetic resonance imaging revealed a reduction in abnormal leptomeningeal enhancement, and the CSF pressure and carcinoembryonic antigen levels were also reduced. The patient continued to respond to afatinib treatment (30 mg once daily) with minimal adverse effects.

**Lessons:**

This is the first case report of clinical improvement after afatinib treatment in a patient with lung adenocarcinoma and leptomeningeal metastasis harboring an EGFR complex mutation (exon19del+K754E), and thus provides a clinical reference for treatment with afatinib of cancers harboring EGFR compound mutations.

## 1 Introduction

The prognosis of patients with non-small cell lung cancer (NSCLC) and leptomeningeal metastasis is poor. The overall incidence of leptomeningeal metastasis in patients with NSCLC has increased in recent years to approximately 3.4% to 3.8%,^[1–3]^ but the incidence is much higher (9.4%) in patients with mutations in the epidermal growth factor receptor (EGFR).^[2]^ The sensitivity of tumors to EGFR tyrosine kinase inhibitors (EGFR-TKIs) is dependent on the specific EGFR gene mutations,^[4]^ and not all patients with EGFR mutations show responses to EGFR-TKIs.^[5]^

We describe here the case of a patient with lung adenocarcinoma and leptomeningeal metastasis harboring an EGFR complex mutation (exon19del+K754E). Treatment with oral afatinib successfully reduced the leptomeningeal metastasis, suggesting that tumors harboring the EGFR exon19del+K754E complex mutation might be sensitive to second-generation EGFR-TKIs.

## 2 Case presentation

A 42-year-old woman with no history of smoking or familial neoplasms presented at the Department of Medical Oncology in February 2018 with a 1-month history of headache, cough, and chest pain. Chest computed tomography (CT) showed a lesion in the upper right lung, suggesting peripheral lung cancer. Enhanced magnetic resonance imaging (MRI) revealed an abnormal enhanced signal in the pons and left occipital lobe, indicating possible intracranial metastasis. CT-guided percutaneous biopsy of the lung tumor and pathological analysis confirmed lung adenocarcinoma. Next-generation sequencing (NGS) of the lung tumor revealed an EGFR exon19 L747_E749del+K754E (c.2260A>G) mutation. The patient began molecular targeted therapy with oral gefitinib (250 mg once daily [qd]), a first-generation EGFR-TKI, and the intracranial metastasis was treated with gamma knife surgery. After 5 months on oral gefitinib, the intracranial lesions progressed. The patient was switched to the third-generation EGFR-TKI osimertinib (80 mg qd). After 2 months on oral osimertinib, the intracranial lesions progressed. She received 2 cycles of combined intravenous chemotherapy and anti-angiogenesis therapy (pemetrexed 800 mg, carboplatin 550 mg, and bevacizumab 550 mg), but the disease continued to progress. The patient was switched to 3 cycles of docetaxel (120 mg) and bevacizumab (500 mg). The patients clinical symptoms continued to deteriorate, with aggravating headache and intermittent loss of consciousness.

**Figure 1:**
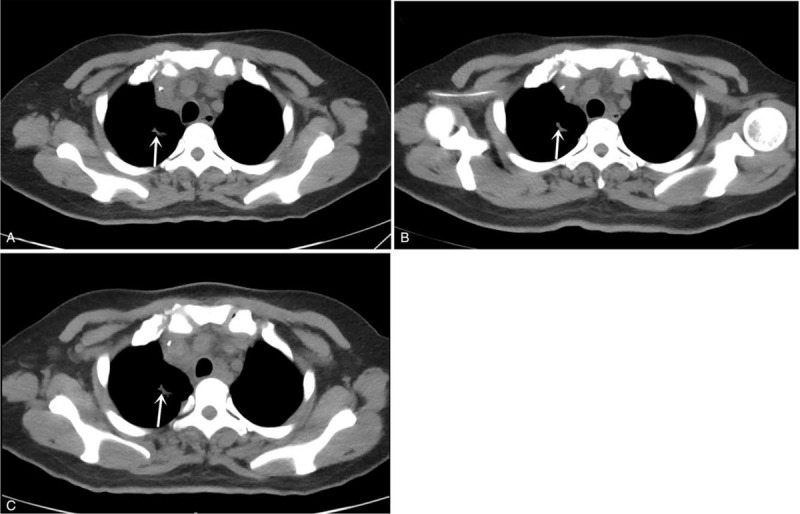
Chest CTs. A-C, Mediastinal window of the chest CT performed on April 01, 2019, showing a mass measuring 10.4 × 4.1 mm in the upper right lung. B, Mediastinal window of the chest CT performed on May 15, 2019, showing a mass measuring 10.3 × 4.0 mm in the upper right lung. C, Mediastinal window of chest CT performed on June 19, 2019, showing a mass measuring 10.3 × 4.0 mm occupying the upper right pulmonary space.

**Figure 2:**
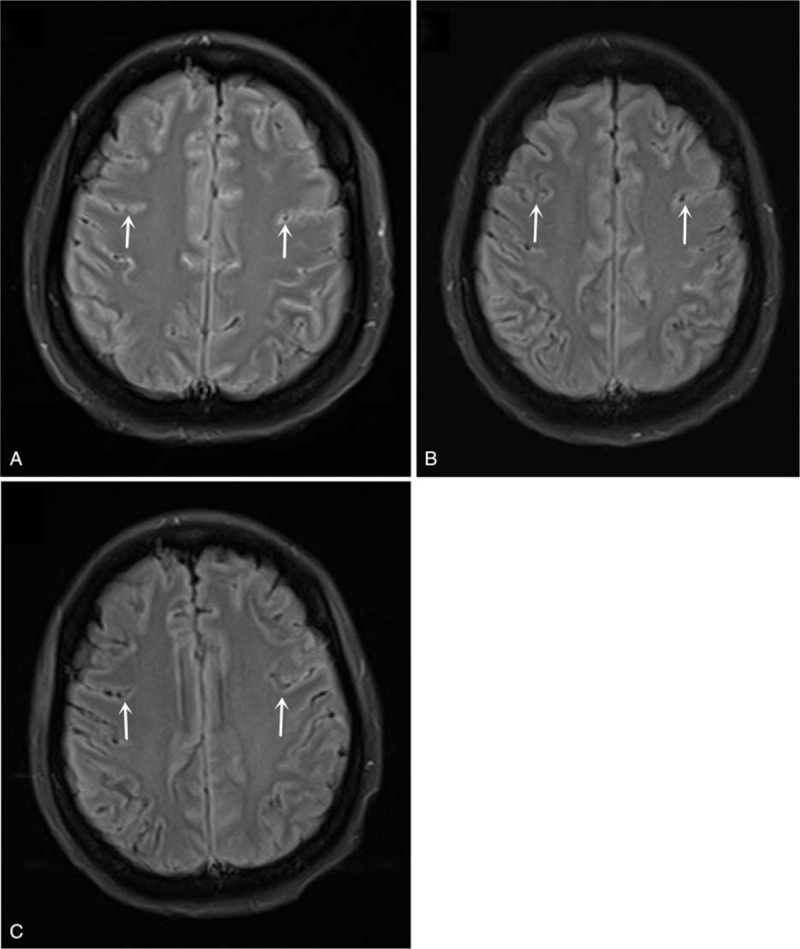
Cranial MRIs. A, Enhanced MRI scan performed on April 01, 2019 with slightly abnormal signal enhancement in the pons, indicative of brain metastasis. Linear FLAIR signals were present around the brainstem, bilateral cerebral hemispheres, and cerebellar sulci, which was indicative of soft meningeal metastasis. B, Enhanced MRI scan performed on May 15, 2019 with linear FLAIR signals around the brainstem, bilateral cerebral hemispheres, and cerebellar hemispheres in the sulci, suggesting soft meningeal metastasis. The range was smaller than that seen on the previous MRI. C, Enhanced MRI scan performed on June 19, 2019 indicating few linear FLAIR signals in the left parietal and right parietal localized sulci. The range was smaller than that seen on the previous MRI.

**Figure 3:**
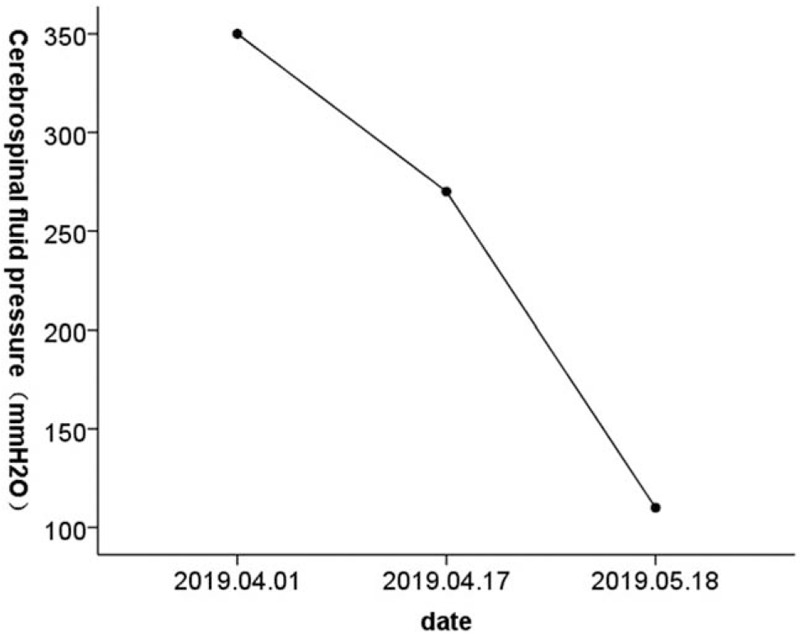
Reduction in cerebrospinal fluid pressure.

**Figure 4:**
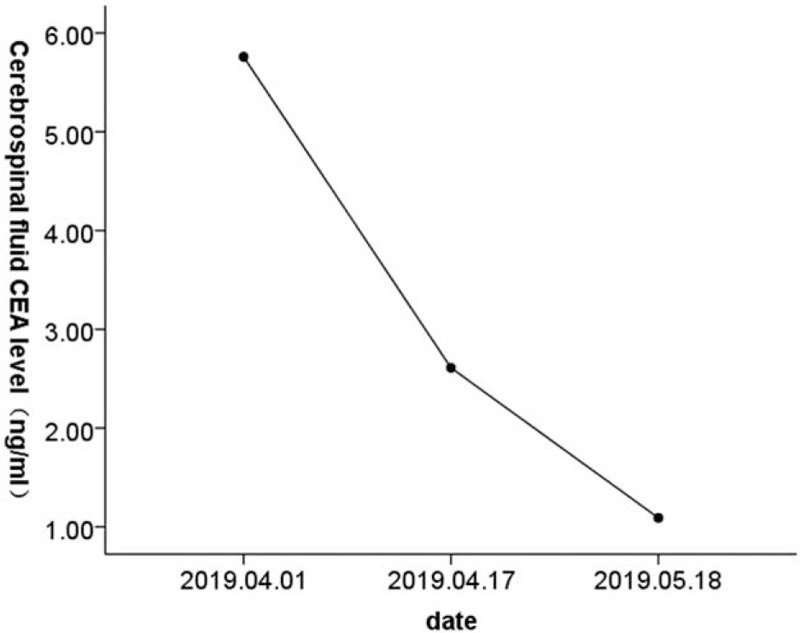
Reduction in cerebrospinal fluid CEA level.

The patient again visited our emergency department and was admitted on March 31, 2019. Chest CT showed a lesion in the upper right lung indicative of lung cancer, with enlarged lymph nodes in the hilus of the right lung, indicating metastasis (Fig. 1A). Enhanced MRI was performed on April 1, 2019, and showed a mild abnormal enhancement in the pons, suggestive of intracranial metastasis. Linear fluid-attenuated inversion recovery (FLAIR) hypersignals around the brain stem in both cerebral hemispheres and within the sulcus of the cerebellar hemisphere were indicative of leptomeningeal metastasis (Fig. 2A). Further diagnosis confirmed peripheral cancer of the right lung (T3N1M1c: brain leptomeninges, lymph node metastasis, stage IV) and the Eastern Cooperative Oncology Group performance status (ECOG PS) was 4. Lumbar puncture was performed on April 1, 2019. The CSF pressure was elevated at 350 mmH_2_O (normal 80–180 mmH_2_O), and the CEA level was 5.76 ng/ml (Figs. 3 and 4). NGS of CSF and peripheral blood samples revealed an EGFR L747_E749 deletion with an abundance of 37.4% and 1.9% in the CSF and blood samples, respectively. The analysis also identified an EGFR K754E (c.2260A>G) mutation with an abundance of 36.4% and 1.9% in the CSF and blood samples, respectively.

On April 10, 2019, the patient started oral afatinib (40 mg po qd), but she developed a grade III oral mucosal reaction 1 week later. The afatinib dose was reduced to 30 mg po qd. A lumbar puncture performed on April 17, 2019, showed a CSF pressure of 270 mmH_2_O and CEA level of 2.61 ng/ml (Figs. 3 and 4). The patient reported headache relief, and she was discharged on April 25, 2019. The patient continued to take afatinib 30 mg po qd at home. The patient again visited our department and was admitted on May 14, 2019. A chest CT performed on May 15, 2019, showed no progression of lung cancer or metastasis (Fig. 1B), and enhanced MRI showed reduced signs of leptomeningeal metastasis (Fig. 2B). A lumbar puncture performed on May 18, 2019, showed a CSF pressure of 110 mmH_2_O and CEA level of 1.09 ng/ml (Figs. 3 and 4). The patient was discharged on May 29, 2019, and continued to take afatinib 30 mg po qd at home. Chest CT and enhanced MRI were performed on June 19, 2019, and showed further reductions in signs of lung and leptomeningeal metastases (Fig. 1C, 2C), consistent with partial remission. The patients headache had also improved, and the ECOG PS was 1. She experienced grade I rash and oral ulcer adverse reactions, but she continued to take afatinib 30 mg po qd at home.

## 3 Discussion

Lung adenocarcinoma is the most common type of NSCLC. EGFR is one of the most frequently mutated driver genes involved in the pathogenesis of NSCLC and is present in 20% and 40% of the Caucasian and Asian patient populations, respectively. EGFR mutations are particularly common in non-smoking Asian female patients with adenocarcinoma.^[5,6]^ EGFR mutations are most frequently located in exons 19 and 21; indeed, exon 19 in-frame deletions and the exon 21 L858R point mutation account for 90% of all EGFR somatic mutations (collectively referred to as classical mutations). Of the remaining (non-classical) mutations, about 6% are complex mutations.^[7,8]^

In the present study, the NGS of a lung tumor biopsy sample identified an EGFR L747_E749del+K754E complex mutation. The patient was treated with gefitinib and then osimertinib, but the intracranial metastasis and clinical symptoms were stable for only 5 and 2 months, respectively. The results of the Phase III FLAURA study (NCT02296125)^[9]^ in patients with newly diagnosed advanced NSCLC and central nervous system metastasis showed a median progression-free survival (PFS) of 13.9 months following treatment with first-generation EGFR-TKIs (gefitinib or erlotinib) and >16.5 months with osimertinib. However, the PFS of our patient after treatment with gefitinib and osimertinib was shorter than the patients in the FLAURA study treated with first- and third-generation EGFR-TKIs. Notably, genetic testing of the patient did not reveal any other driver mutations that could account for drug resistance. We, therefore, speculate that the lack of effect of gefitinib and osimertinib in our patient may be due to the presence of the non-classical mutation K754E. A search using the molecular analysis tool PolyPhen-2 (http://genetics.bwh.harvard.edu/pph2/) indicated that EGFR p.K754E is a potential damaging mutation (position-specific independent count [PSIC], 0.607; sensitivity, 0.80; specificity, 0.83). Thus, there is a need to identify drugs with efficacy against tumors carrying the EGFR p.K754E mutation. The LUX-Lung3 and LUX-Lung6 clinical trials showed that afatinib improved the clinical symptoms and PFS in patients with NSCLC harboring classical and non-classical EGFR mutations.^[10]^ The LUX-Lung2, LUX-Lung3, and LUX-Lung6 clinical trials in patients with advanced NSCLC harboring non-classical mutations also showed responses to afatinib. Based on these studies, afatinib was approved by the US Food and Drug Administration to treat metastatic NSCLC with non-classical EGFR mutations.^[11,12]^

Ma et al^[13]^ reported on the efficacy of afatinib treatment in a patient with NSCLC and a non-classical EGFR mutation (G719A). Frega et al^[8]^ have also reported on the response to afatinib treatment of a patient with NSCLC carrying 3 non-classical mutations: exon 18 E709K and an exon 21 L833V_H835L complex mutation. These findings prompted us to treat our patient with afatinib upon identification of the EGFR 19del+K754E mutation. After 1 week on afatinib, the patients headache was improved, although she also developed grade I rash and grade III oral mucosa reactions. The patient continued to take afatinib (30 mg po qd) for 1 month, and enhanced MRI performed 2 months later revealed reduced enhancement of the leptomeningeal metastasis, with persistently stable pulmonary lesions. The patients headache continued to improve, and the CSF pressure and CEA level were both reduced. These findings are consistent with suppression of leptomeningeal metastasis, and we conclude that the EGFR p.K754E non-classical mutation may be associated with the response to afatinib treatment.

## 4 Conclusion

To the best of our knowledge, the current study represents the first case report of a response to afatinib treatment in a patient with lung adenocarcinoma and leptomeningeal metastasis harboring an EGFR L747_E749del+K754E complex mutation. This report should serve as a clinical reference for the use of afatinib to treat tumors harboring non-classical mutations.

## Acknowledgments

The authors thank the bioinformatics team at Livzongene LLC for their assistance. We thank Anne M. O’Rourke, PhD, from Liwen Bianji, Edanz Group China (www.liwenbianji.cn/ac), for editing the English text of a draft of this manuscript.

## Author contributions

MC and LM collected the data and drafted the manuscript. JR performed the statistical analysis and participated in its design. MN, LJ, and LL assisted in drafting the manuscript. All authors read and approved the final manuscript.

**Conceptualization:** Rong Jiang.

**Data curation:** Chunhua Ma, Mei Liu.

**Formal analysis:** Rong Jiang.

**Project administration:** Rong Jiang.

**Resources:** Ning Mu, Jinduo Li, Lin Li.

**Writing – original draft:** Chunhua Ma, Mei Liu, Ning Mu, Jinduo Li, Lin Li, Rong Jiang.

**Writing – review & editing:** Chunhua Ma, Mei Liu, Rong Jiang.
